# GFAP variant p. Tyr366Cys demonstrated widespread brain cavitation in neonatal Alexander disease.

**DOI:** 10.1016/j.radcr.2021.11.066

**Published:** 2021-12-28

**Authors:** Hirokazu Takeuchi, Norimichi Higurashi, Hiroshi Kawame, Tadashi Kaname, Kumiko Yanagi, Yuichiro Nonaka, Tatsuya Hirotsu, Satoshi Matsushima, Tetsuya Shimizu, Taku Gomi, Nei Fukasawa

**Affiliations:** a Department of Pediatrics, Jikei University School of Medicine Tokyo, Japan, 3-25-8, Nishi-Shimbashi, Minato-ku, Tokyo, 105-8461; b Department of Clinical Genetics, Jikei University School of Medicine Tokyo, Japan, 3-25-8, Nishi-Shimbashi, Minato-ku, Tokyo, 105-8461; c Department of Genome Medicine, National Center for Child Health and Development, Japan, 2-10-1 Okura, Setagaya-ku, Tokyo; d Department of Neurosurgery, Jikei University School of Medicine Tokyo, Japan, 3-25-8, Nishi-Shimbashi, Minato-ku, Tokyo, 105-8461; e Department of Radiology, Jikei University School of Medicine Tokyo, Japan, 3-25-8, Nishi-Shimbashi, Minato-ku, Tokyo, 105-8461; f Department of Pathology, Jikei University School of Medicine Tokyo, Japan, 3-25-8, Nishi-Shimbashi, Minato-ku, Tokyo, 105-8461

**Keywords:** Alexander disease, Cavitation, GFAP, Magnetic resonance imaging, White matter

## Abstract

Alexander disease is a rare form of leukodystrophy caused by heterozygous mutations in the gene encoding glial fibrillary acidic protein (GFAP). Brain cavitation in the white matter, predominantly distributed in the frontal periventricular area, has been described in some cases. Here, we present a case of a 1-year-old boy with neonatal Alexander disease caused by the p. Tyr366Cys *GFAP* variant, with rapid and widespread white matter cavitation. This case broadens the radiological spectrum of Alexander disease and suggests a possible genotype-phenotype correlation between the p. Tyr366Cys variant and cavitation.

## Introduction

Alexander disease (AxD) is a rare form of leukodystrophy caused by heterozygous mutations in the gene encoding glial fibrillary acidic protein (GFAP). GFAP is an intermediate filament protein expressed in astrocytes [1]. Abnormal GFAP accumulation due to disease-causing *GFAP* mutations leads to astrocyte dysfunction [2] and the formation of cytoplasmic protein aggregates called “Rosenthal fibers” (RFs). RFs are histopathological indicators of AxD, as are small heat shock proteins and alpha B-crystallin.

AxD is clinically classified into three age-related subtypes: neonatal or infantile, juvenile, and adult [3]. Disease severity decreases as the age of onset increases [4]. Neonatal AxD was first described in 2000 [5,6] and is characterized by aqueductal stenosis-induced congenital hydrocephalus, white matter abnormalities, intractable seizures, severe psychomotor impairments, and increased cerebrospinal fluid (CSF) protein concentration [5,6].

Magnetic resonance imaging (MRI) criteria of AxD were proposed in 2001, which included abnormalities of the white matter, especially in the frontal area, hyperintense T1 signal and hypointense T2 signal of the periventricular rim, abnormalities of the brainstem, thalami, and basal ganglia, and enhancement of brain structures [7]. Brain cavitation in AxD is an uncommon finding of white matter abnormalities and is described as a cystic white matter degeneration clearly identifiable in approximately one-third of patients [7,8]. Cavitation is observed most frequently in the periventricular area [5,^7^,8]. However, further characteristics are unknown.

Here, we present a case of neonatal AxD where cavitation appeared at an early phase and extended subcortically in a short period. We also discuss a possible genotype-phenotype correlation.

## Case report

**Fig. 1:**
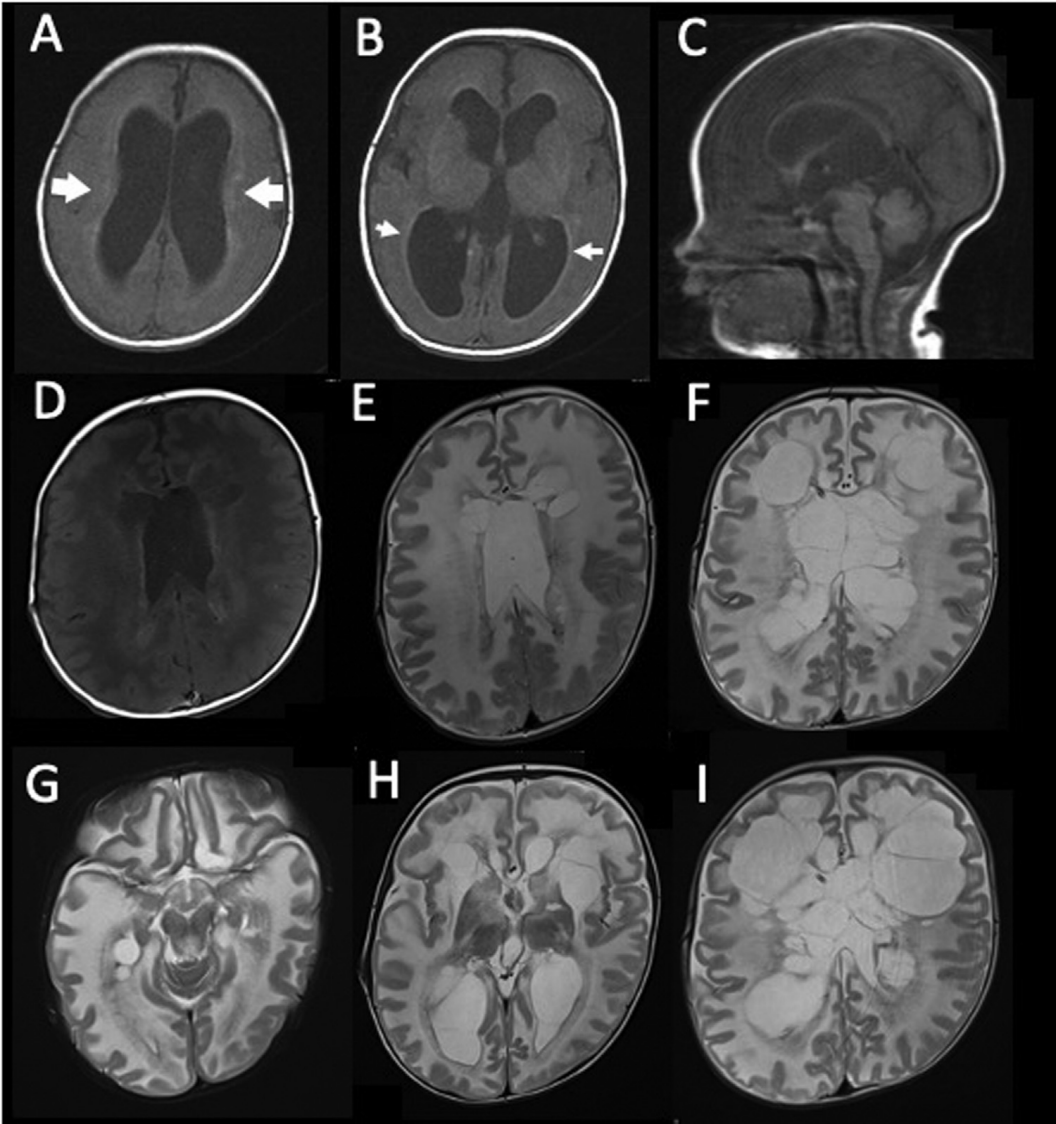
Magnetic resonance imaging of the brain. (A–C), T1-weighted images 2 days after birth. (A), the lateral ventricles are enlarged, and a hyperintense signal in the deep white matter is observed (large white arrow). (B), a hyperintense signal classified as periventricular rim is shown (small white arrow). (C), the sagittal image shows a narrow aqueductus cerebri. (D and E), images at 5 months of age. (D), the T1-weighted image shows a hyperintense signal in the deep white matter and cavitation localized to the frontal area. (E), the T2-weighted image shows a diffuse hyperintense signal in the white matter. (F–H), T2-weighted images at 10 months of age. (F), cystic transformation of the white matter extends to the occipital area. (G), a hyperintense signal in the brainstem is shown. (H), atrophy of the thalamus is observed. (I), the T2-weighted image at 13 months shows further extension of the cavitation

A 1-year-old boy presented with ventriculomegaly at 36 weeks of gestation and was born at 39 weeks of gestation. His birth weight, body length, and head circumference were 3085 g (69.1 percentile), 50.7 cm (82.1 percentile), and 36.5 cm (>98 percentile), respectively. His muscle tone was normal, and no dysmorphic features other than macrocephaly were identified. Brain MRI obtained 2 days after birth showed a narrow aqueductus cerebri, ventriculomegaly, and abnormal signal intensity in the white matter (Fig. 1A–C). A ventriculoperitoneal (VP) shunt was inserted at 14 days of age. Protein levels in the ventricular CSF were persistently high, and shunt revisions were required at 3, 4, and 6 months of age due to repeated tube obstruction. Brain MRI at 5 months of age revealed severe hypomyelination and periventricular cavitation localized to the frontal area (Fig. 1D, E).

The patient's psychomotor development was significantly impaired. At 5 months of age, he was unable to control his neck, vocalize, or track objects. At 6 months of age, he developed focal clonic seizures involving the left arm and eyelid, which were controlled by levetiracetam. During follow-up, limb hypertonia and gastroesophageal reflux were observed.

**Fig. 2:**
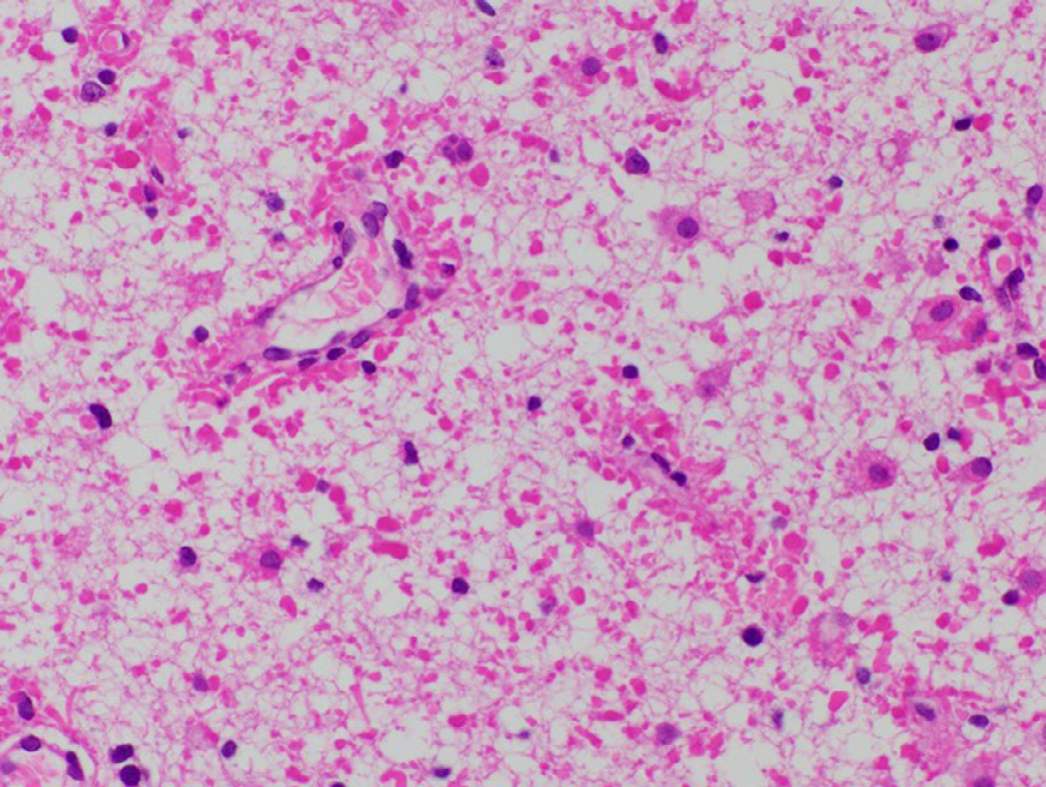
Histopathological findings. Hematoxylin and eosin staining of frontal white matter shows hypomyelination and accumulation of oval-shaped Rosenthal fibers, especially on the astrocyte end-feet around blood vessels

At 10 months of age, the circumference of his head increased. Brain MRI showed an exacerbation of the ventriculomegaly and widespread cystic degeneration (Fig. 1F–H). The protein level in ventricular CSF sampled via the shunt tube was 1202 mg/dL. Cyst fenestration was performed to reduce intracranial pressure. The frontal brain tissue biopsied intraoperatively revealed hypomyelination and RF accumulation predominantly in the white matter (Fig. 2).

Whole exome sequencing was performed after receiving informed consent from the patient's parents. Whole exome sequencing of blood DNA revealed a de novo heterozygous missense mutation in exon 6 of *GFAP* (c. 1097A→G; p. Tyr366Cys). Since this variant has been associated with the infantile-onset of AxD, a diagnosis of neonatal AxD was made. At 14 months of age, the patient was bedridden with spastic quadriplegia. Brain MRI showed further progression of the white matter cavitation (Fig. 1I). This study was approved by the ethical committee of the National Center for Child Health and Development. We obtained permission for the publication from the patient's parents.

## Discussion

We present a case of neonatal AxD that exhibited significant cavitation with frontal predominance involving a large area of the white matter. Protein levels in the CSF were persistently high, and multiple VP shunt revisions were required because of repeated tube obstruction. The patient had an AxD-associated missense variant in the *GFAP* gene (p. Tyr366Cys) [8].

The appearance of cavitation is correlated with early-onset [7,9], with the youngest case showing cavitation in MRI at the age of 10 months [8]. The location of the cavitation was localized to the periventricular area, frontal in most cases [5,[7], [8], [9]. In the present case, MRI showed cavitation at 5 months of age and rapidly extended to the subcortical areas. Excessive accumulation of GFAP, which elicits structural changes that disrupt gap junctions and synapse in astrocytes, resulting in neuronal cell degeneration, is a potential cause of cavitation [1].

GFAP levels in the CSF correspond to GFAP levels in the brain [10] and tend to be higher in early-versus late-onset cases [11]. The GFAP levels in the CSF were not obtained in the present case. However, the onset suggests that the GFAP levels may have been high in the brain. Since the GFAP levels in the CSF vary depending on the *GFAP* variant [12], a relationship may exist between cavitation and the genotype.

An association between the *GFAP* variants and clinical and radiological phenotypes has been reported in several studies [3,4]. Two missense *GFAP* variants, namely R79 and R239, are frequently observed in neonatal AxD and correlate with the frontal predominance of white matter abnormalities and basal ganglia/thalamus signaling dysfunction [4]. The *GFAP* variant in the 2B domain, a highly conserved and functionally important component [13], tends to present with an early onset and a severe phenotype [3]. Although cases of cavitation in AxD have been reported, the causative genetic variant was identified as p. Tyr366Cys in only one case [8]. Therefore, p. Tyr366Cys may have a relationship with brain cavitation. Further genetic research on AxD with cavitation is required.

In our case and others, hydrocephalus due to aqueductal stenosis, periventricular rim in the brain MRI, and elevated CSF protein levels were observed in the early phase of neonatal AxD [14,15]. Aqueductal stenosis is the result of GFAP accumulation in the subependymal area and brainstem and the consequent proliferation of astrocytes in the brainstem [16]. It is recommended to check for the periventricular rim, a specific feature of neonatal AxD when hydrocephalus due to narrow cerebral aqueducts and elevated CSF protein levels are observed in infants. AxD is untreatable at present. However, these indicators may be useful in predicting the disease and preventing its progression in the future.

We present a case of neonatal AxD with rapidly expanding cavitation potentially caused by the p. Tyr366Cys *GFAP* variant. This case broadens the radiological spectrum of AxD and suggests a possible relationship between significant cavitation formation and p. Tyr366Cys.

In cases of congenital hydrocephalus due to aqueductal stenosis and elevated CSF protein levels, it is clinically important to suspect neonatal AxD before the appearance of cavitation and to carefully check for the periventricular rim in brain MRI images.

## Competing interests

The author has no conflicts of interest to disclose concerning the presentation.

## Patient Consent Statement

Written informed consent for publication was obtained from the patient's parents.
